# Evolution of effective serial interval of SARS-CoV-2 by non-pharmaceutical interventions

**DOI:** 10.21203/rs.3.rs-32486/v1

**Published:** 2020-06-01

**Authors:** Sheikh Taslim Ali, Lin Wang, Eric H. Y. Lau, Xiao-Ke Xu, Zhanwei Du, Ye Wu, Gabriel M. Leung, Benjamin J. Cowling

**Affiliations:** The University of Hong Kong; University of Cambridge; The University of Hong Kong; Dalian Minzu University; University of Texas at Austin; Beijing Normal University; The University of Hong Kong; The University of Hong Kong

**Keywords:** SARS-CoV-2, COVID-19, serial interval, transmission, control, public health

## Abstract

Studies of novel coronavirus disease (COVID-19) have reported varying estimates of epidemiological parameters such as serial intervals and reproduction numbers. By compiling a unique line-list database of transmission pairs in mainland China, we demonstrated that serial intervals of COVID-19 have shortened substantially from a mean of 7.8 days to 2.6 days within a month. This change is driven by enhanced non-pharmaceutical interventions, in particular case isolation. We also demonstrated that using real-time estimation of serial intervals allowing for variation over time would provide more accurate estimates of reproduction numbers, than by using conventional definition of fixed serial interval distributions. These findings are essential to improve the assessment of transmission dynamics, forecasting future incidence, and estimating the impact of control measures.

## Background

In December 2019, a novel coronavirus disease 2019 (COVID–19), caused by severe acute respiratory syndrome coronavirus 2 (SARS-CoV–2), was first reported in Wuhan, China, and has since spread to more than 212 countries, causing more than 4.6 million confirmed cases and 300,000 deaths worldwide by 18 May 2020 (1). Recent studies suggest that several demographic and social factors can influence the transmission of COVID–19, including age or gender-related difference in infection risk (2–4), reduced risk of infection due to intensive non-pharmaceutical interventions (NPIs) (e.g., isolation, social distancing) (5–7), and abrupt changes in social mixing patterns due to lockdowns and confinement (8–10). Serial interval, defined as the duration between the symptom onset time of infector and that of the infectee, is an essential metric for estimating many other key epidemiological parameters (e.g., reproduction number, generation time, and attack rate), which are in turn used to predict disease trends and healthcare demands (11). In early studies before availability of specific data on COVID–19, the serial interval distribution of COVID–19 was assumed to be similar to that of Severe Acute Respiratory Syndrome or Middle East Respiratory Syndrome, with a mean longer than 8 days (12, 13). Once specific data became available on COVID–19 transmission pairs, a number of studies have examined the serial interval distribution of COVID–19 in different locations, with estimates of the mean serial interval varying from 3.1 days to 7.5 days (6, 14–21). All of these studies assumed that the timing of transmission events can be described by a single stable distribution of serial intervals at different stage of an epidemic.

In fact, the serial interval depends on the incubation period and the profile of infectiousness after infection. The incubation period describes the biological process of disease progression and should tend to follow a more similar distribution from one location to another, with minor differences perhaps due to social or cultural differences in how symptoms are perceived or reported. However, the profile of infectiousness over time can vary because of human behavior. Changes in contact patterns and the use of public health measures can potentially reshape the timing of infection events by limiting successful contacts overall (e.g., social distancing) or after illness onset (e.g., case isolation). Interventions such as the isolation of confirmed and suspected cases, suspension of intra- and inter-city travel, and different forms of social distancing were widely implemented in different Chinese cities. This provides a unique opportunity to study the temporal changes in the serial interval distribution and its association with NPIs. Here, we show that variation in the serial interval can occur, and has important implications on the assessment of transmission dynamics and the impact of control measures.

## Results

### Transmission pairs and serial intervals

We compiled a database of 1,407 COVID–19 transmission pairs, in which symptom onset dates and social relationship were available for both the infector and infectee of 679 transmission pairs (see Table S1 for entire database, and supplementary materials section 1 and 2 for more data descriptions). Household and non-household transmissions were identified from the information of social relationships (e.g., familial members of the same household, non-household relatives, colleagues, classmates, friends, and other face-to-face contacts). The data were reconstructed from the publicly-available reports of 9,120 confirmed COVID–19 cases reported by 27 provincial and 264 urban health commissions in China outside Hubei province. Data from Hubei province were excluded because there was less reliable information on chains of transmission during widespread community circulation of COVID–19, whereas outside Hubei province it was more straightforward to link connected cases and derive serial intervals. We focused on 677 transmission pairs with infectors developed symptom from January 9 through February 13, 2020. This 36-day period covers a series of key events related to the evolving epidemiology and transmission dynamics of COVID–19 in mainland China (22–24).

**Figure 1:**
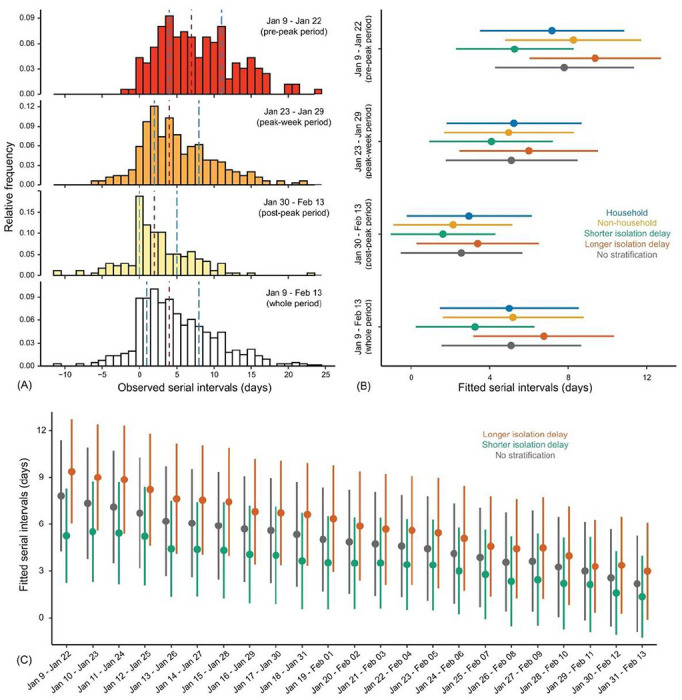
Evolution of serial intervals of COVID-19 in mainland China. (A) Empirical serial interval distributions. In top three panels, the infector of each transmission pair developed symptom during January 9 22, 2020 (pre-peak period), January 23 29, 2020 (peak-week period), and January 30 February 13, 2020 (post-peak period), respectively. In the bottom panel, the infectors developed symptom during the whole 36-day period. In each panel, vertical dashed lines in red and blue colors indicate the median and interquartile range (IQR). (B) Estimated serial interval distributions by fitting a normal distribution to serial interval data via MCMC. From top to bottom, each group of bars correspond to transmission pairs with infectors developed symptom during the pre-peak, peak-week, post-peak, and whole 34-day period, respectively. Colored dots and bars correspond to the transmission pairs within households (blue), outside of households (yellow), with isolation delay shorter than the median isolation delay of each period (green), and with isolation delay longer than the median isolation delay of each period (orange), respectively. Dark-grey bars correspond to all transmission pairs (no stratification). (C) Estimated serial interval distribution for each running time window by fitting a normal distribution. Dark-grey color indicates fitting serial interval data with no stratification, whereas green (yellow) indicates fitting data with isolation delay shorter (longer) than the median isolation delay of each running time window. In (B) and (C), dots and bars indicate the estimated median and IQR, respectively.

We first calculated the number of transmission pairs in our database by the onset dates of infectors (fig. S2). Since a large number of infectors (339, ~50% of data) developed symptom during January 23–29, 2020, we defined this 1-week period as the peak-week period, the earlier14-day period (January 9–22, 2020) as the pre-peak period, and the later 15-day period (January 30–February 13, 2020) as the post-peak period. We computed the serial interval as the number of days between the symptom onset dates of the infector and that of the infectee for each transmission pair. Empirical serial interval distributions for transmission pairs with infectors developed symptom during each of these 3 successive and non-overlapping periods suggest that the serial intervals were substantially shortened overtime (Fig. 1A)..

We then estimated the serial interval distribution during each non-overlapping period by fitting a normal distribution to the corresponding serial intervals data (see Supplementary Materials section 3). In analysis of the entire dataset of 677 transmission pairs, we estimated that the serial interval distribution had a mean of 5.1 (95% credibility interval, CrI: 4.7, 5.5) days and standard deviation of 5.3 (95% CrI: 5.0, 5.6) days (table S2), which are consistent with recent studies (16, 21, 25). However, fitting to data of non-overlapping subsets revealed considerable variation in serial interval distributions overtime (Fig. 1B).. The mean and standard deviation of serial intervals were estimated to be 7.8 (7.0, 8.6) days and 5.2 (4.7, 5.9) days during the pre-peak period, reduced to 5.1 (4.6, 5.7) days and 5.0 (4.6, 5.4) days during the peak-week period, and be further shortened to 2.6 (1.9, 3.2) days and 4.6 (4.2, 5.1) days during the post-peak period, respectively (table S2).

Next, we examined the evolution of transmission pairs using a series of running time windows with fixed length of 10, 14 or 18 days (fig. S3). In stark contrast to the use of a single stable distribution of serial intervals, our analysis suggests that the serial intervals were gradually shortened over the study period (Fig. 1C)„ which is robust against alternative specifications of time windows (fig. S3). By fitting the transmission pairs data of each running time-window via Markov Chain Monte Carlo (MCMC) method (Fig. 1C and table S3), we estimated that during the first 14-day period (January 9–22, 2020) the serial intervals were longer on average (mean: 7.8 (95% CrI: 7.0, 8.6) days, and standard deviation (sd): 5.2 (95% CrI: 4.7, 5.9) days), whereas during the last 14 days (January 30–February 13, 2020) the serial intervals were much shorter on average (mean: 2.2 (1.5, 2.9) days, and sd: 4.6 (4.1, 5.1) days). Notably, the mean serial intervals were shortened by more than threefold over the 36-day period.

**Figure 2:**
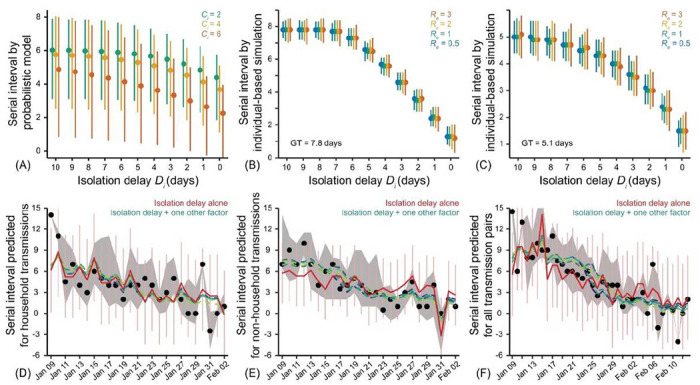
Effect of non-pharmaceutical interventions (NPI) on shortening serial intervals over time. (A) Serial intervals estimated with the probabilistic model of transmission pair, in which the start time of infectiousness C_i is considered as 2 (green), 4 (yellow), or 6 (orange) days before symptom onset of infector. Given each isolation delay D_i, the dot and vertical bar indicate the mean and interquartile range (IQR) of estimated distribution. (B)-(C) Serial intervals estimated by the individual-based simulation model. Given each combination of isolation delay D_i and initial effective reproduction number R_e, the dots and vertical bars indicate the median and IQR of estimated distribution, when the mean generation time (GT) is (B) 7.8 days and (C) 5.1 days. (D)-(F) Prediction of empirical serial intervals with infectors that developed symptom on each day (by multivariable regression models), for (D) household, (E) non-household, and (F) all transmission pairs. Black dots and grey shaded regions indicate the median and IQR of empirical serial intervals. Red curves indicate the mean (with 95% confidence interval in light-pink vertical bars) of serial intervals predicted with the basic regression model only accounting for the isolation delay of infectors. Dashed curves in other colors indicate the mean serial intervals predicted by extending the basic regression model to further account for any one of the following factors: isolating confirmed cases, isolating suspected cases, inter-city travel ban, intra-city travel ban, closure of public services, closure of entertainment venues, social mobilization and accumulation of population immunity (see supplementary materials, section 6, for details).

In our data of 677 transmission pairs, the information of age, sex, household, and isolation delay (i.e., time duration from symptom onset to isolation) is available for most infectors. This allows a granular stratification. Using either non-overlapping or running time windows for data stratified by each of these factors, we find a similar decreasing pattern of serial intervals overtime (Fig. 1, B and C, tables S2 and S3). Therefore, we termed this evolving serial interval overtime as the “effective serial interval”, which accounts for temporal changes due to the effects of its potential driving factors. Notably, the length of effective serial intervals is positively associated with the length of isolation delay (Figs. 1C and 2A to C, tables S3 and S4), accounting for the decreasing pattern of isolation delay overtime (fig. S4). Considering all 677 transmission pairs together, early isolation (shorter than the median isolation delay) is associated with shorter serial intervals (mean: 3.3 (2.7, 3.8) days, and sd: 4.5 (4.1, 4.9) days), and delayed isolation (longer than the median isolation delay) is associated with a longer serial interval (mean: 6.8 (6.2, 7.3) days, and sd: 5.3 (4.9, 5.7) days) (table S2). Stratification by age, gender or household shows no clear difference in serial interval estimates. Our findings are robust against using alterative distributions (e.g. Gumbel distribution) for model fitting (fig. S5).

### Effect of non-pharmaceutical interventions on shortening effective serial intervals over time

To understand the influence of isolation delay, we first used a probabilistic model of transmission pairs to analyze the effect of reducing the time delay between illness onset and isolation of the infector on serial interval distribution. This model considers that the infector has a time-varying infectiousness that starts Ci days before symptom onset, reaches a peak around the time of illness onset, and then declines thereafter (Supplementary Materials section 4). We parameterized the start time Ci and infectiousness profile with published data (20). This simple model suggests that serial intervals tend to be shortened with reduced time delay in isolating infector, regardless of when infector starts to be infectious before illness onset (Fig. 2A)..

To further examine the association between serial interval and isolation delay, we simulated serial intervals using an individual-based model that tracks the infection and symptom onset times of each case (Supplementary Materials section 5, Fig. 2, B and C, and table S4). Given a mean generation time of 7.8 days (as per our estimate for the pre-peak period of COVID–19 in mainland China), the simulated mean serial intervals reduces from ~8.0 to ~1.2 days when the isolation delay reduces from 10 to 0 days. Given a mean generation time of 5.1 days (as per our estimate for the peak-week period of COVID–19 in mainland China, which is similar to the estimates by Zhang. et. al. (25)), the simulated mean serial interval reduces from ~5.1 to ~1.5 days when isolation delay reduces from 10 to 0 days. Similar outcomes were obtained with alternative generation times with a mean of 2.6 days (estimate for the post-peak period) and 8.4 days (as estimated for the 2003 SARS epidemic (26)) (table S4). The evolution of serial intervals is less sensitive to the change in initial effective reproduction number, Re (a measure of initial transmission accounting for the effect of control measures). The above analytical and simulated models validate that serial interval is positively associated with isolation delay.

Since the implementation of a cordon sanitaire around Wuhan on January 23, 2020, multiple NPI strategies have been implemented in more than 260 Chinese cities, including the isolation of confirmed and suspected cases, suspension of intra-city public transport, suspension of travel between cities, social distancing by closure of entertainment and public gathering venues (e.g., bar, cinema, park) as well as public services (e.g., shopping malls, restaurants), and recruitment of governmental staff and volunteers to enforce quarantine (fig. S6). As the pandemic unfolds, the accumulation of population immunity may also alter the risk of infection overtime (27–29). To study the influence of these factors on COVID–19 transmission, we developed a series of linear multivariable regression models to predict empirical serial intervals with infectors that developed symptom on each day (Supplementary Materials section 6). The basic regression model only accounting for isolation delay can explain up to 51.5% of variability in daily empirical serial intervals, indicating isolation delay as the prime factor. The improved models that combine the basic model with one of the additional factors (NPI strategy or accumulation of population immunity) can explain a further maximum of 15.6% - 20.3% variability in daily empirical serial intervals (table S5). The model fitting further suggests a potential explanation about how serial intervals can be modulated by respective interventions over the span of outbreak (Fig. 2D to F).. We found that, per day of early isolation, the predicted serial interval decreased by 0.7 (95% confidence interval, CI: 0.4, 0.9) days on average. Although the effects of these additional factors in combination of isolation delay are identified specifically in non-household setting, we were not able to detect their individual contribution to change serial intervals (table S5).

### Real-time transmissibility estimated with a single stable serial interval distribution versus effective serial interval distributions

The real-time transmissibility of an infectious disease is often characterized by the instantaneous reproduction number (*R_t_*), which is defined as the expected number of secondary infections caused by an infector on day *t*. The epidemic is capable to spread when *R_t_*>1 and under control when *R_t_*<1. To estimate *R_t_*, a routine protocol is to approximate the generation time distribution with a single stable serial interval distribution. Let *w_i_* be the serial interval distribution that approximates the infectiousness profile of an infected individual at *i*-th day since infection. Then, the daily estimate of

$$R_{t}=\frac{I_{t}}{\sum_{i=1}^{t}I_{t-i}w_{i}}$$

is calculated as the ratio between the number of cases It on day *t* and the weighted average of infectiousness caused by cases infected before day *t*,

$$\sum_{i=1}^{t}I_{t-i}w_{i}.$$

**Figure 3:**
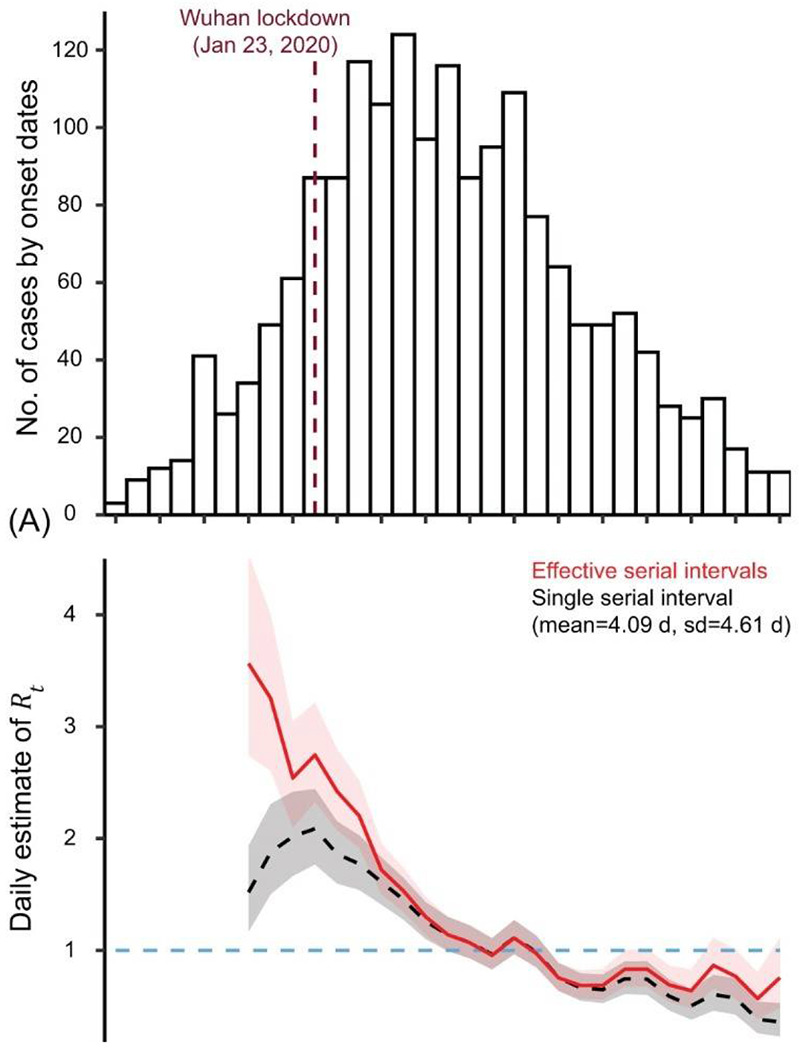

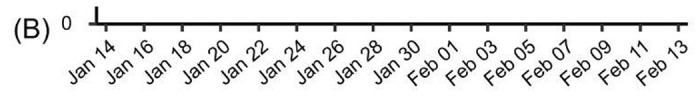
Daily estimate of the instantaneous reproduction number (R_t) by using a single stable serial interval distribution and time-varying effective serial interval distributions. (A) Daily number of COVID-19 cases by symptom onset dates, based on our transmission pairs data. Dashed dark-red line indicates the start of Wuhan lockdown. (B) Daily R_t estimated from the epi-curve data in (A). Dashed black curve indicates the daily median R_t estimated by assuming a single stable serial interval distribution (mean = 4.09 days, sd = 4.61 days) for the whole 36-day period. Red curve indicates the daily median R_t estimated with time-varying effective serial interval distributions. Grey and light-pink shaded regions indicate the 95% CrI of R_t estimated with a single stable or time-varying serial interval distribution, respectively.

To examine the effect of serial intervals, we first obtained the daily number of cases based on the onset dates of infectors and infectees in our entire database of 1,407 transmission pairs (Fig. 3A). Then using the statistical method developed by Cori et al (30), we estimated *R_t_* for each day between January 20 and February 13, 2020. We noticed a substantial difference in estimates of *R_t_* between using a single stable serial interval distribution and time-varying effective serial interval distributions. The magnitude of this difference depends on the discrepancy between the single fixed serial interval distribution and the time-varying serial interval distributions, and is more prominent during the pre-peak and post-peak periods compared to that of during the peak week when *R_t_*≈1 (Fig. 3 and fig. S7).

## Discussion

The serial intervals of COVID–19 in mainland China were reduced by more than threefold across the 36-day period (January 9–February 13, 2020). The continuous reduction in serial intervals overtime was driven by intensive non-pharmaceutical interventions. The combination of analytical, simulation, and regression models validates that the reduced isolation delay is the prime factor shortening serial intervals over time. We estimated that serial intervals would be shorten by more than 3 days, for those pairs for which infectors were isolated faster compared to that of isolated later from their first symptoms (Fig. 1B to C, tables S2 and S3). Isolation of an infector one day earlier is expected to reduce the mean serial interval by 0.7 days on average. This is consistent with the advocate that isolating cases and quarantining their contacts within 1 day from symptom onset may reduce COVID–19 transmission by 60% (8).

The regression model accounting for isolation delay alone can explain up to 51.5% of the variance in observed serial intervals. Further inclusion of the factors on population immunity or any NPI strategy improves the model performance in explain the variance in serial interval. However we are not able to disentangle the individual roles of these factors on evolving serial intervals, as most of these measures were initiated within the same week across cities (fig. S6). We have not identified any significant role of gender and age of infectors except some temporal variations for serial intervals, although it has been reported that the SARS-CoV–2 circulated more in older adults than young adults and children (3, 31).

Our study demonstrates that serial intervals are highly dependent on the timeliness of case isolation. Other studies (15, 20) estimate that the transmissibility of COVID–19 was considerably high before and immediately after the symptom onset. Although the short serial interval indicates that a substantial proportion of transmission events have already occurred around the time of symptom onset (14), prolonged viral shedding allows a wider opportunity window for spreading the disease (14, 32, 33) and suggests for extended isolation. While general preventive measures (e.g., enhanced personal hygiene or social distancing for both pre-symptomatic and symptomatic cases) may not have a significant impact on serial intervals, specific preventive measures that limit the duration of potential transmissions (e.g., isolation of illness or more intensive contact tracing and quarantine) is expected to compress the serial interval. An overall change in observed serial intervals provides a straightforward signal that indicates successful implementation of these measures. Therefore, the temporal variation in serial intervals may also indicate potential areas for improving epidemic parameter estimation and its consequent applications.

Our study has some limitations. First, there could be recall bias on the onset of first symptoms in the line-list data. Considering the centralized pandemic response in mainland China, we expect that the recall bias would not affect our main conclusions. Second, the shortening effective serial intervals over time could have also been influenced by factors other than those considered here. We explained up to 72% of the variance in observed serial intervals, and the remainder remains unexplained. Third, the multivariable regression models can only estimate the combined effect, and they were not able to disentangle the probable non-linear effects of these factors and potential confounder with serial intervals. Finally, data on transmission pairs may not always be readily available over the course of an epidemic, especially at the initial stage.

Our results suggest that caution is needed when attempting to generalise estimates of the serial interval distribution from one location to another, for example when estimating instantaneous reproductive numbers (Fig. 3 and fig. S7). The real-time metric of effective serial intervals suggests that transmission models need to account for the temporal variation in serial intervals through an epidemic, specifically for emerging infectious diseases. The effective serial intervals may provide better measurement of instantaneous transmissibility (Rt) by including the effects of possible drivers of transmission, and be helpful to policy makers to have real-time information on the impact of public health measures. The positive association between isolation delay and effective serial interval recommends early contact tracing and isolation as one of the feasible strategies for controlling COVID–19, which calls for new studies to quantify and optimize such measures.

## Supplementary Material

Supplement

