# Supplementary material for: Evolution of effective serial interval of SARS-CoV-2 by non-pharmaceutical interventions
Source: Res Sq. 2020 Jun 1:rs.3.rs-32486. Preprint. [Version 1] doi: 10.21203/rs.3.rs-32486/v1 (PMC7336698; doi:10.21203/rs.3.rs-32486/v1)
Supplement: Supplement [file Alisupplement20may.docx]

Supplementary Materials for

Evolution of effective serial interval of SARS-CoV-2 by non-pharmaceutical interventions

Sheikh Taslim Ali^1,†^ , Lin Wang^2,†^, Eric HY Lau^1,†^, Xiao-Ke Xu^3^, Zhanwei Du^4^, Ye Wu ^5,6^, Gabriel M. Leung^1^, Benjamin J. Cowling^1,*^

^*^ **Correspondence to:** bcowling@hku.hk

**This PDF file includes:**

Materials and Methods

Supplementary Text

Figs. S1 to S7

Tables S1 to S5

**Table of Contents**

|  |  | Pages |
| --- | --- | --- |
| 1. | **Reconstruction of transmission pairs** | 3 |
| 2. | **Stratifications of transmission pairs** | 5 |
| 3. | **Estimation of serial interval** | 6 |
| 4. | **Probabilistic model of transmission pair** | 6 |
| 5. | **Individual-based model for simulating serial intervals** | 9 |
| 6. | **Multivariable regression model** | 10 |
| 7. | **References** | 22-23 |

Materials and Methods

1. **Reconstruction of transmission pairs**

In mainland China, 27 provincial and 264 urban health commissions have publicly posted detailed reports of 9,120 confirmed COVID-19 cases online since 20 January 2020. As of 19 February 2020, cases with detailed reports comprise 72.7% of all COVID-19 cases confirmed in China outside Hubei Province. The original case reports include the demographic information (e.g., age, gender, residential city), travel history (e.g., dates of entry and exit), exposure history if known (e.g., the infector who caused this infectee and their social relationship), and epidemiological timelines (e.g., potential time of infection, symptom onset, hospital visit(s), isolation, confirmation, and disclosure). Below is an exemplary transmission pair identified from the original public disclosures posted online, which is translated from Chinese to English:

*Case XZ-1:* Mr. X, male. He is from Chongqing and works in Guangzhou. He took a train from Guangzhou on 13 January 2020 and arrived at Wuhan’s Hankou railway station in the morning of14 January 2020. He stayed in a hotel in Wuhan for 5-6 hours, and then transferred at noon on the 14th, he took the K1068 train from Hankou to Xuzhou. He arrived in Xuzhou at 20:52 on the same day. Fever, dizziness and other discomfort occurred in the afternoon of 19 January. On the same day, he went to Quanshan District Peace Community Health Service Center for infusion treatment. On the morning of January 23, he went to the fever clinic of the Affiliated Hospital of Xuzhou Medical University and the chest radiograph showed the lung with textured thickening and patchy shadows. Now he has been transferred to Xuzhou Medical University Hospital (East Hospital) for isolation and treatment. The case was confirmed on 26 January and is in stable condition. (The above information was announced by Xuzhou Health Commission on January 27.)

*Case XZ-4:* Ms. Y, female. She is the eldest daughter of patient Xuzhou-1. She had been living in Quanshan district of Xuzhou city for 2 weeks before the onset of the disease. No history of exposure to Wuhan, but close contact with the confirmed case Xuzhou-1. On 24 January, she developed symptoms of fever, sneezing and runny nose. She took oseltamivir orally by herself and did not attend the hospital. The case was confirmed on 27 January. On the same day, she went to Quanshan District Peace Community Health Service Center for infusion treatment. On the morning of January 23, she went to the fever clinic of the Affiliated Hospital of Xuzhou Medical University and the chest radiograph showed the lung with textured thickening and patchy shadows. Now she has been transferred to Xuzhou Medical University Hospital (East Hospital) for quarantine and treatment. The case is in stable condition. (The above information was announced by Xuzhou Health Commission on January 28.)

*Case XZ-1* is a male family member who travelled to Wuhan on 13-14 January 2020 and returned home in Xuzhou on 14 January 2020. *XZ-1* developed symptom on 19 January 2020, went to hospital on 23 January 2020, and was confirmed as a COVID-19 case on 26 January 2020. *Case XZ-4* is the daughter of Case *XZ-1*. She did not have travel history to Wuhan, but used to stay at the same household with the Case *XZ-1* after his return. She developed symptoms on 24 January 2020 and was later confirmed on 27 January 2020.

Given a transmission pair of infector and infectee, the infector denotes the *primary* case and the infectee denotes the *secondary* case. From the public disclosure data of 9,120 confirmed COVID-19 cases, we reconstructed 1,407 transmission pairs with strong evidence that the infectee was infected by the infector. Each transmission pair is established according to the travel history, social relationship, and epidemiological timelines, which were cross-validated by three co-authors. If an infectee had multiple possible exposures, the infector of this transmission pair is considered as the one reported the earliest symptom onset.

1. **Stratifications of transmission pairs**

Each transmission pair is categorized by social relationship between the infector and infectee (e.g., familial members within the same household, non-household relatives, colleagues, classmates, friends, and other face-to-face contacts). The online reports do not directly state whether the family members live in the same household. As an approximation, we assumed any transmission pair among grandparents, parents and children as a *household* transmission pair. Other than *household* transmission pair, it is termed as *non-household* transmission pair.

We stratified these transmission pairs by the following settings. Denote the *isolation delay* as the time duration from the symptom onset to isolation time of the infector for each transmission pair. The s*horter* (*longer*) *isolation delay* is used to find the transmission pairs with isolation delays that are shorter (longer) than the median isolation delay across all transmission pairs identified with a given condition of stratification (e.g., household transmission). The *younger-age* (*older-age*) are used to find the transmission pairs with infectors that are younger (older) than the median age across all infectors of transmission pairs identified with a given condition (e.g., household transmission). The indicator *male* and *female* are defined similarly.

1. **Estimation of serial interval**

Given a transmission pair, the serial interval is computed as the number of days between the reported symptom onset date of the infector and of the infectee. Serial interval distribution is estimated by fitting a normal (or Gumbel) distribution to the corresponding data via Markov Chain Monte Carlo (MCMC) method with Gibbs sampling and non-informative flat prior. To fit a normal distribution, we estimated the mean and standard deviation of the normal distribution; to fit a Gumbel distribution, we estimated the location and scale parameters of the Gumbel distribution. The convergence of MCMC chains is confirmed using trace plot and diagnosis. The posterior distribution of parameters is obtained by running 100,000 iterations with a burn-in of 40,000 iterations and a thinning interval of 10.

1. **Probabilistic model of transmission pair**

Given a transmission pair in which the infectee *j* is infected by the infector *i* at time $T_{j}^{I}$, the serial interval denotes the time duration between the symptom onset time of infector $T_{i}^{ES}$ and symptom onset time of infectee $T_{j}^{ES}$: $SI_{i,j}=T_{j}^{ES}-T_{i}^{ES}$.

Denote $T_{i}^{EA}$ as the time at which the infector *i* starts to be infectious, $T_{i}^{ISO}$ the time at which the infector *i* is isolated, and $D_{i}=T_{i}^{ISO}-T_{i}^{ES}$ the time delay from symptom onset to isolation of infector *i*, respectively. We term $D_{i}$ as the isolation delay.
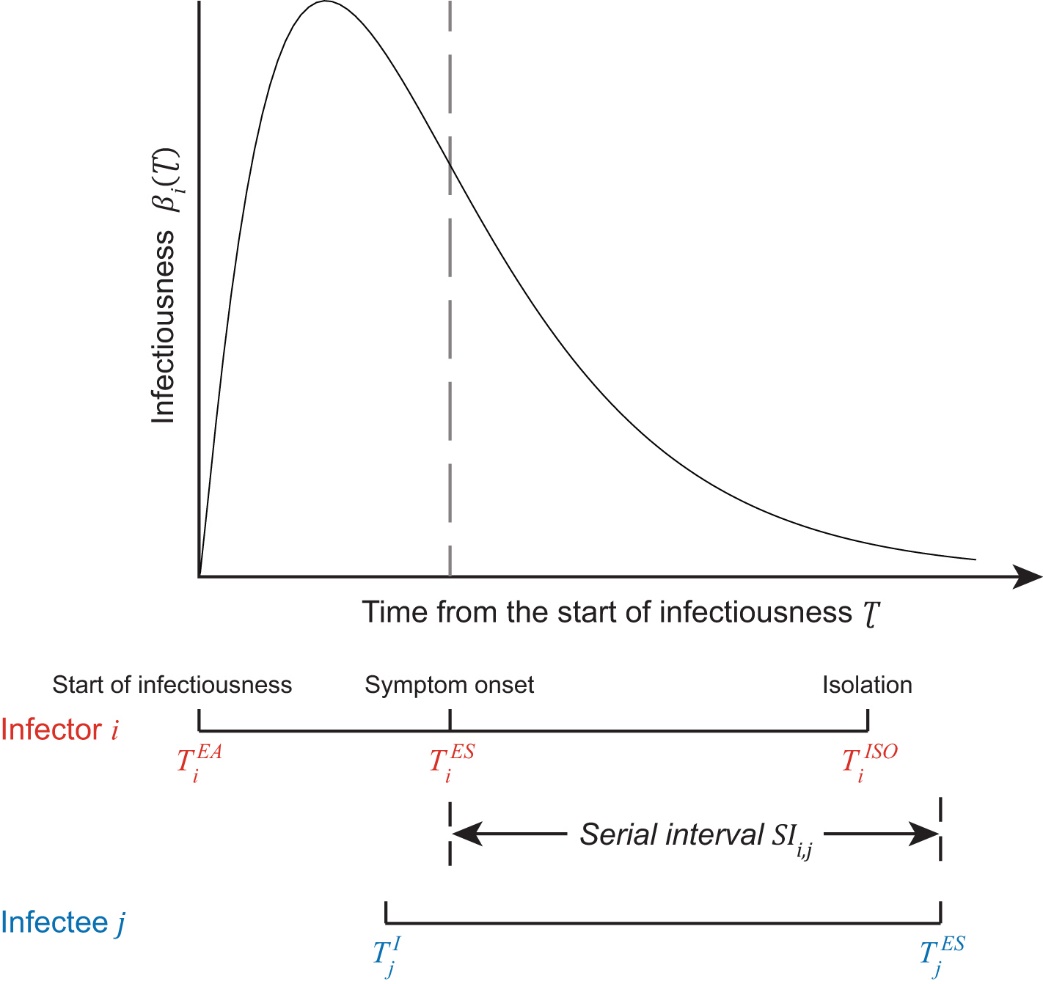


*Fig. S1. Schematic of a transmission pair. The infector* i *starts to be infectious and symptomatic, and then is isolated at time* $\mathbf{T}_{\mathbf{i}}^{\mathbf{EA}}$*,* $\mathbf{T}_{\mathbf{i}}^{\mathbf{ES}}$*, and* $\mathbf{T}_{\mathbf{i}}^{\mathbf{ISO}}$*, respectively. The infectee* j *is infected by the infector* i *at time* $\mathbf{T}_{\mathbf{j}}^{\mathbf{I}}$ *and becomes symptomatic at time* $\mathbf{T}_{\mathbf{j}}^{\mathbf{ES}}$*. Top panel illustrates the infectiousness profile of infector* i*, which is assumed to follow a gamma distribution.*

Following He et al. [1], we define the infectiousness of infector *i* as the probability to infect a infectee $\tau=T_{j}^{I}-T_{i}^{EA}$ days after infector *i* starts to be infectious at time $T_{i}^{EA}$. The probability density function (pdf) of infectiousness is assumed to follow a gamma distribution [1]: $\beta_{i}\left( \tau\right)=\frac{1}{\Gamma\left( k \right)\cdot\theta^{k}}\cdot\tau^{k-1}\cdot\exp\left( -\frac{\tau}{\theta} \right)$, where $k$ and $\theta$ are the shape and scale parameters, respectively. Recent clinical [2-4], epidemiologic [1, 5-8], and biological studies [9-11] suggest that the SARS-CoV-2 infection is able to shed virus particles before symptom onset, which causes a substantial proportion of pre-symptom transmissions. Hence we consider that the infectiousness starts from $C_{i}$ days before symptom onset of infector *i*, i.e., $C_{i}=T_{i}^{ES}-T_{i}^{EA}$. Based on these settings, the expression of serial interval becomes:

$$SI_{i,j}=E_{j}^{S}+\tau-C_{i}$$

where the first term $E_{j}^{S}=T_{j}^{ES}-T_{j}^{I}$ is the incubation period of infectee *j*. Based on the detailed exposure history for the first 425 confirmed SARS-CoV-2 cases in Wuhan, Li et al. [12] suggested that the pdf of incubation period $P(E^{S})$ follows a log-normal distribution with a mean of 5.22 days and standard deviation of 3.87 days.

Given an isolation delay $D_{i}$ that is assumed to occur before symptom onset of infectee, the pdf of serial intervals is obtained by integrating over the range of time interval $\tau$:

$$P\left( SI_{i,j} \right)=\int_{0}^{C_{i}+D_{i}} \beta_{i}\left( \tau\right)\cdot P\left( E^{s}=SI_{i,j}+C_{i}-\tau\right)d\tau$$

The cumulative distribution is given by $\int_{-\infty}^{x} P\left( SI_{i,j} \right)dSI_{i,j}$, and the mean serial interval is given by: $\int_{-\infty}^{\infty} SI_{i,j}P\left( SI_{i,j} \right)dSI_{i,j}$.

To compare with data of transmission pairs with observed serial interval and isolation delay, we computed the pdf of serial intervals with minimum isolation delay$D_{i}^{min}$:

$$P\left( SI_{i,j},D_{i}^{min} \right)=\int_{D_{i}^{min}}^{\infty} P(D_{i})\int_{0}^{C_{i}+D_{i}} \beta_{i}\left( \tau\right)\cdot P\left( E^{s}=SI_{i,j}+C_{i}-\tau\right)d\tau dD_{i}$$

where $P(D_{i})$ is the distribution of isolation delay $D_{i}$, which is obtained by fitting a log-normal distribution to isolation delay data across all transmission pairs.

We parameterized the start time of infectiousness $C_{i}$, and the shape $k$ and scale $\theta$ of the gamma distribution of infectiousness with inferred results by He, X. et al. [1]. Specifically, we tested the following 3 scenarios which were selected from the high likelihood region estimated by He, X. et al. [1].

| Parameters | Scenario-I | Scenario-II | Scenario-III |
| --- | --- | --- | --- |
| $C_{i}$ | 2 days | 4 days | 6 days |
| Shape $k$ | 2.15 | 2.74 | 1.53 |
| Scale $\theta$ | 1.34 | 1.71 | 4.38 |

1. **Individual-based model for simulating serial intervals**

We simulated the serial intervals by using an individual-based model. Specifically, we started with a number of cases who have been infected at the time origin. Each potential infector will infect a number of infectees, which follows a Poisson distribution with mean$R_{e}$, the effective reproduction number. The exact infection times will be simulated according to the generation time. However, knowledge on the generation time for COVID-19 is still limited, and hence was approximated by published serial intervals, with a gamma distribution and a mean of 5.1 days [14] and also same as our estimates during peak-week period for COVID-19 in mainland China. In other scenarios, we assumed short and long serial intervals respectively, which were the same as that estimated with means of 7.8 (as estimated from the pre-peak period), 2.6 (as estimated from the post-peak period) and 8.4 days (as estimated for the 2003 SARS epidemic (26)). From the simulated infection times, we added the simulated incubation period for all infectees, assuming a lognormal distribution with mean 5.2 days [12]. The duration between the symptom onset times of the infector and infectee in each transmission pair would be the simulated serial intervals. We present the mean serial intervals and their 95% confidence intervals from 500 transmission pairs in 200 simulations under different initial effective reproduction number $R_{e}$ (e.g. 3.0, 2.5, 2.0, 1.5, 1.0, 0.5, 0.3) [15] to represent situations with different level of general control measures (e.g. social distancing, enhanced personal hygiene) in place, and reducing time delay from symptom onset to isolation (e.g. from 10 to 0 days) which was assumed to interrupt further transmission after a case is isolated.

1. **Multivariable regression model**

Our analysis suggests that the real-time effective serial intervals can be significantly driven by the time delay in isolating infector. Notably, the effective serial intervals were shortened by more than threefold over the 34-day period from 9 January 2020 to 13 February 2020. Aside from the case isolation, other non-pharmaceutical interventions (NPI) including the travel ban and social distancing, and the accumulation of population immunity due to the depletion of susceptible people may also contribute to declining the effective serial interval.

We evaluated the daily time series of following measures from transmission pairs. Denote, $\omega(t)$ and $d(t)$ as the observed serial interval and isolation delay on day$t$, respectively. We calculated $\omega(t)$ by the median of all serial intervals with infectors developed symptoms on day$t$, and $d(t)$ by the median of time delay from symptom onset to isolation for infectors with symptoms during day$t$, who caused infectees further. To quantify the daily intensity of each NPI strategy in mainland China, we also counted the number of cities in mainland China that implemented each NPI measure on day$t$, using the interventions data from more than 260 Chinese cities [16] (fig. S4). The NPI measures include the isolation of suspected and confirmed cases, travel ban for intra-city and inter-city movement, social distancing by closing public services (e.g., hospitals, shopping malls, restaurants) and entertainment venues (e.g., cinema, bar, café) and recruiting government workers and volunteers to assist quarantine and social distancing. Denote $\eta_{k}(t)$ as the cumulative number of cities in mainland China that implemented the $k$-th intervention ($k=1, 2, \ldots, 7$) on day$t$. The accumulation of population immunity is approximated by the cumulative number $\eta_{8}(t)$ of confirmed COVID-19 cases with symptom onset occurred no later than day *t*.

We first used univariate linear regression models to test the association between each intervention measure and daily serial interval$\omega(t)$, and then significant factors are included one in addition to linear multi-variable regression model to predict the daily serial interval $\omega(t)$:

$\omega\left( t \right) \sim\beta_{0}d\left( t \right)+\beta_{k}\eta_{k}\left( t \right)$, $k=1, 2, \ldots, 8$

where each $\beta_{i}$ is a regression coefficient for respective factors. Because the isolation delay is expected to be a prime driver of serial intervals, we considered a baseline model as$\omega_{base}\left( t \right)=\beta_{0}d\left( t \right)$, where $d\left( t \right)$ is the daily isolation delay (the median daily isolation delay). $\eta_{1}\left( t \right)$ and $\eta_{2}\left( t \right)$ are the daily cumulative number of cities implemented the isolation on suspected and confirmed cases respectively. The travel restrictions consist of $\eta_{3}\left( t \right)$ and$\eta_{4}\left( t \right)$, the daily cumulative number of cities implemented the inter-city and intra-city travel ban respectively. The social distancing measures are$\eta_{5}\left( t \right)$, $\eta_{6}\left( t \right)$ and$\eta_{7}\left( t \right)$, the daily cumulative number of cities implemented the closure of public services, closure of entertainment venues, and recruitment of government workers and volunteers to assist quarantine, respectively. Finally, we estimated the contribution of each factor according to the improvement in predicting the daily serial interval $\omega(t)$ by including each factor into the baseline model.


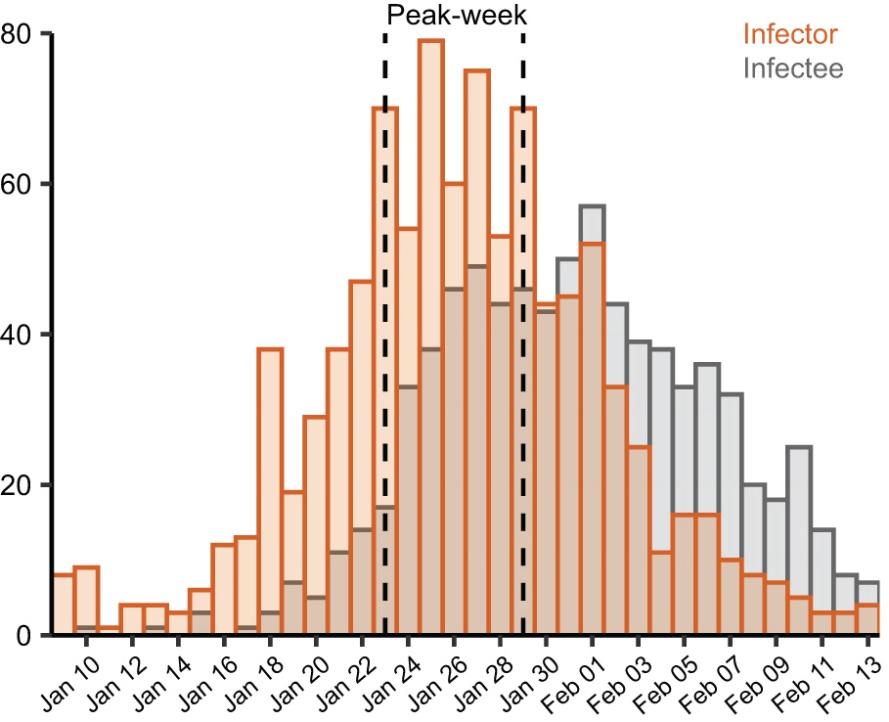


**Fig. S2**: Daily number of infectors (in orange bar) and infectees (in grey bar) by their symptom onset of COVID-19 in China outside Hubei province during January 9 – February 13, 2020. The 1-week period during January 23 – 29, 2020, is regarded as the peak-week period, because a large number of infectors (339, ~50% of data) developed symptom during this week. The earlier14-day period (January 9–22, 2020) is regarded as the pre-peak period, and the later 15-day period (January 30 – February 13, 2020) is regarded as the post-peak period.

**
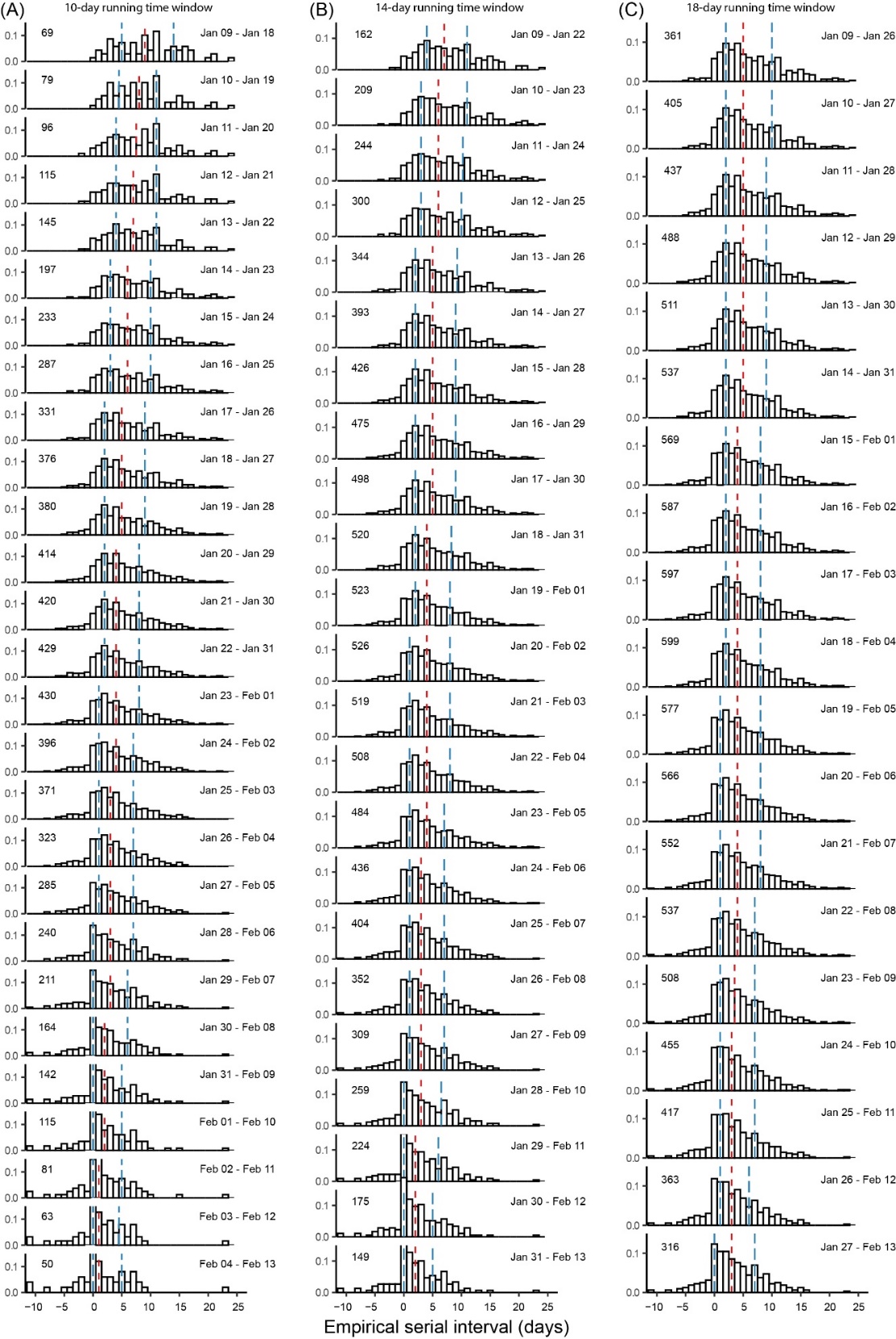
**

**Fig. S3**: Empirical distributions of serial interval data in each running time window shifting from January 9 to February 13, 2020. From left to right, each column corresponds to the use of running time windows of (A) 10-day, (B) 14-day, and (C) 18-day, respectively. In each panel, the number on the upper-left corner indicates the number of transmission pairs with infectors developed symptom during that time window (i.e., sample size *n*).


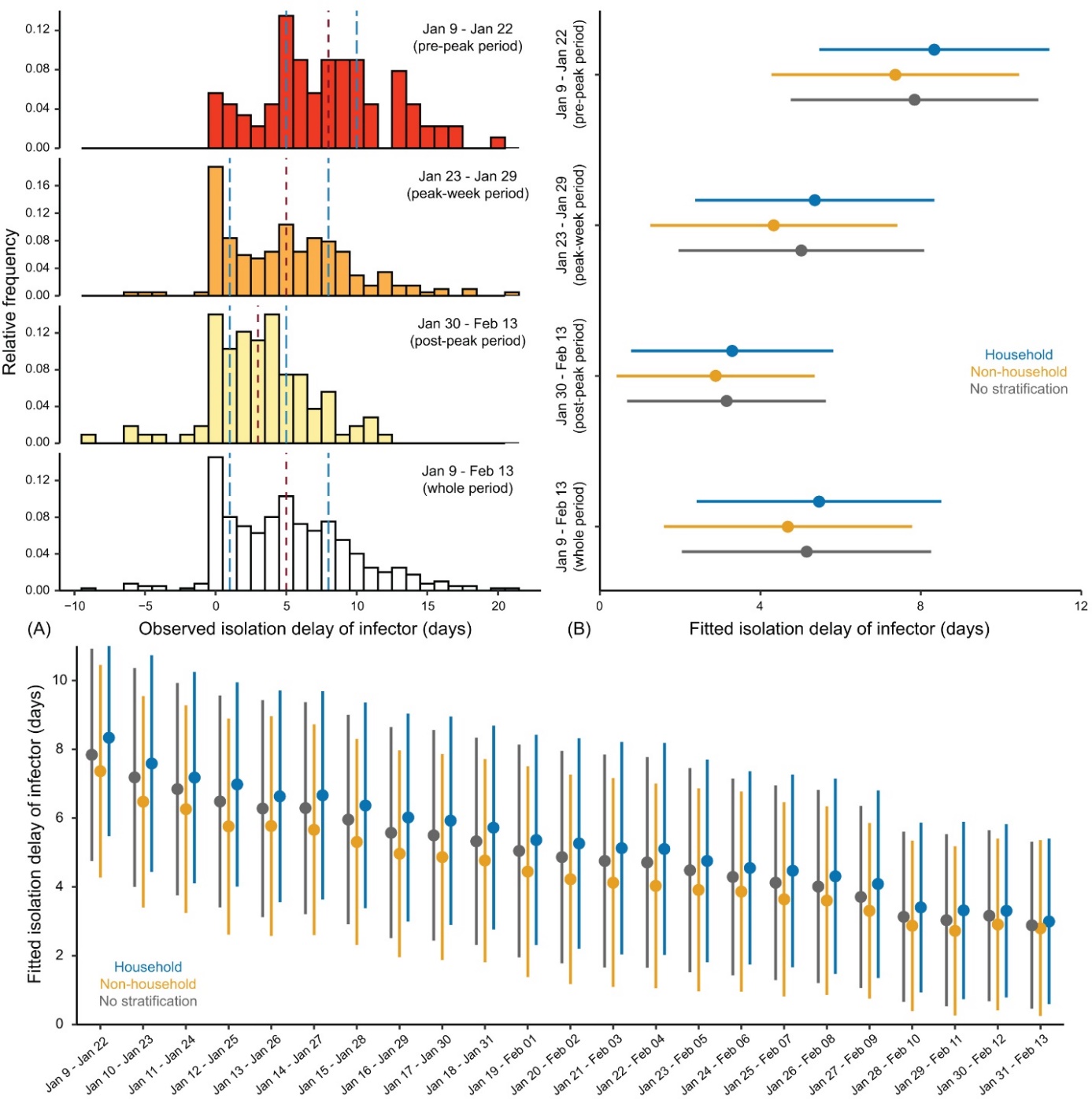


**Fig. S4**: Evolution of time delay in isolating COVID-19 infectors from their symptom onset (i.e., isolation delay) in mainland China. (A) Empirical isolation delay distributions. In top three panels, the infector of each transmission pair developed symptom during January 9 ̶ 22, 2020 (pre-peak period), January 23 ̶ 29, 2020 (peak-week period), and January 30 ̶ February 13, 2020 (post-peak period), respectively. In the bottom panel, the infectors developed symptom during the whole 36-day period. In each panel, vertical dashed lines in red and blue colours indicate the median and interquartile range (IQR). (B) Estimated isolation delay distributions by fitting a normal distribution to isolation delay data via MCMC. From top to bottom, each group of bars correspond to transmission pairs with infectors developed symptom during the pre-peak, peak-week, post-peak, and whole 34-day period, respectively. Coloured dots and bars correspond to the transmission pairs within households (blue), outside of households (yellow), and all transmission pairs with no stratification (dark-grey). (C) Estimated isolation delay distribution for each running time window by fitting a normal distribution. Dark-grey colour indicates fitting data with no stratification, whereas blue (yellow) indicates fitting household (non-household) data. Dots and bars in (B) and (C) indicate the estimated median and IQR, respectively.


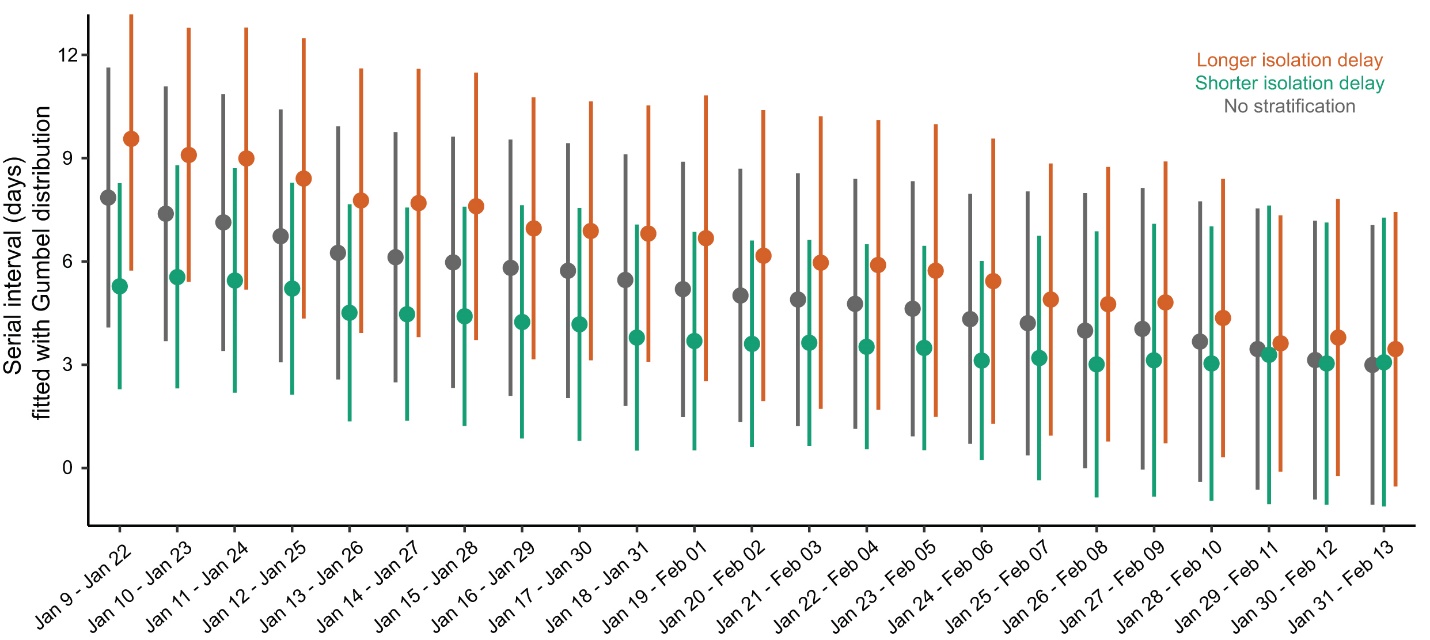


**Fig. S5**: Serial interval distribution estimated for each of the 14-day running time windows. The first running time window contains the transmission pairs with symptom onset of infectors during January 9 – 22, 2020; the second window contains the transmission pairs with symptom onset of infectors during January 10 – 23, 2020; and so on. Posterior samplers are obtained by fitting a Gumbel distribution to the serial interval data in each time window via MCMC. Dots and bars indicate the median and interquartile range (IQR) for the estimated distribution, respectively. Dark-grey colour indicates the fitting for serial interval data with no stratification, whereas green (orange) indicates the fitting for the data with isolation delay shorter (longer) than the median isolation delay of each running time window.


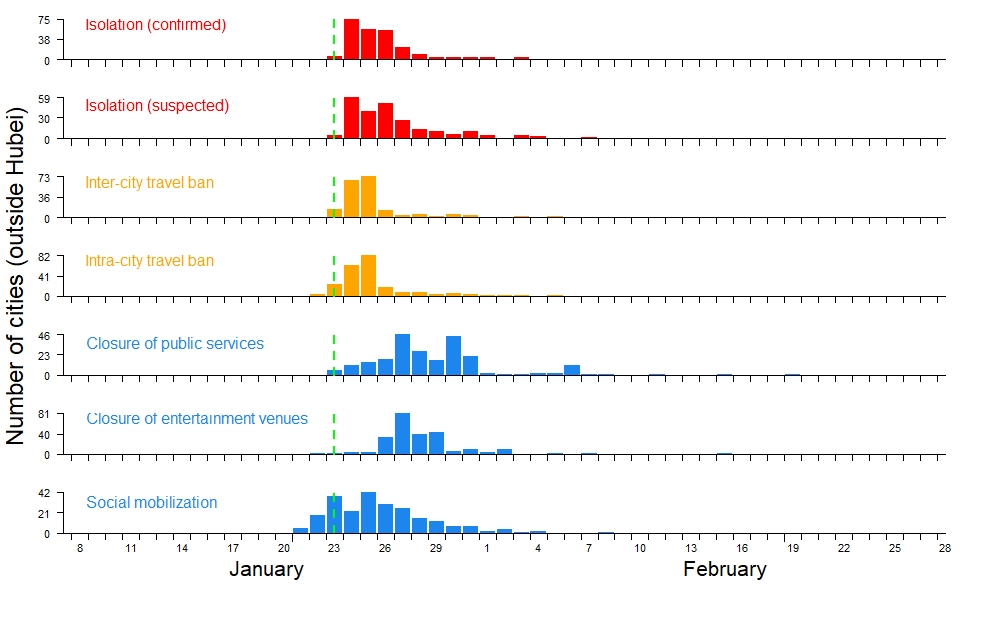


**Fig. S6**: Timelines for the number of Chinese cities (excluding cities in Hubei province) that were starting to implement each of the following 7 non-pharmaceutical interventions: isolation of confirmed cases, insolation of suspected cases, suspension of travel between cities (i.e., inter-city travel ban), suspension of intra-city public transport (intra-city travel ban), closure of public services (e.g., shopping malls, restaurants), closure of entertainment and public gathering venues (e.g., bar, cinema, park), and recruitment of governmental staff and volunteers to enforce quarantine (i.e., social mobilization).


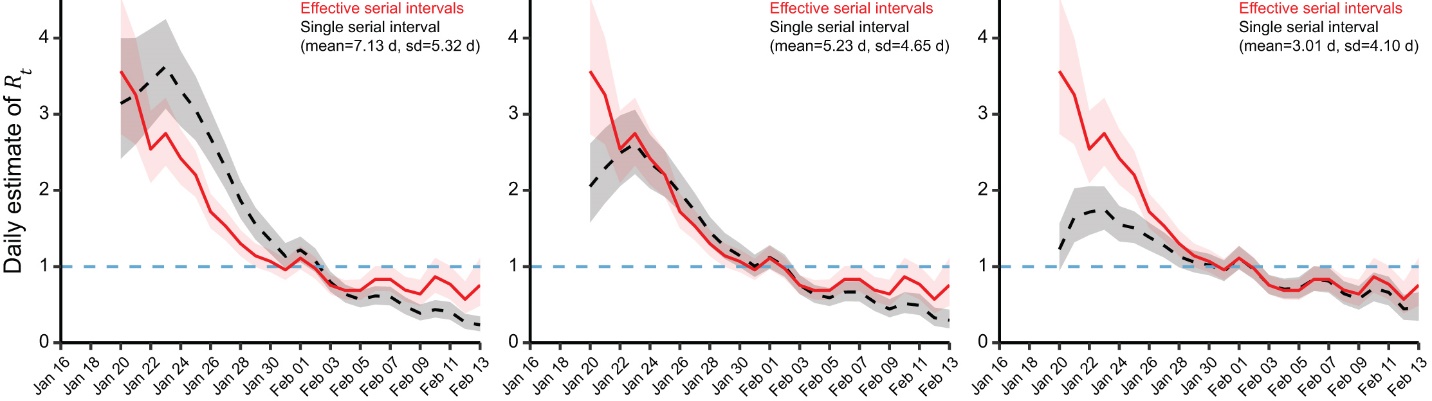


**Fig. S7:** Comparison of instantaneous reproduction number ($R_{t}$) estimated with time-varying effective serial interval distributions and with a single stable serial interval distribution. In all three panels, red lines indicate the median $R_{t}$ estimated with time-varying effective serial interval distributions. Black dotted lines indicate the median $R_{t}$ estimated with a single serial interval distribution with (A) mean=7.1 days and sd=5.3 days, (B) mean=5.2 days and sd=4.7 days (C) mean=3.0 days and sd=4.1 days. Light-grey and light-pink shaded regions indicate the 95% CrI of $R_{t}$ estimated with time-varying and a single serial interval distributions, respectively.

**Table S1**: **Entire dataset of 1,407 transmission pairs in mainland China.**

For reviewing purposes, we uploaded this dataset to the figshare, which is accessible via this link:

<https://figshare.com/s/6d1d23102eb3b092f651>

The dataset will be publicly available at the time of publication.

**Table S2**: Estimated mean and standard deviation (sd) of serial intervals. The columns entitled “Pre-peak period”, “Peak-week period”, “Post-peak period”, and “Whole period” correspond to fitting transmission pair data with infectors developed symptom during the pre-peak period (January 9 ̶ 22, 2020), peak-week period (January 23 ̶ 29, 2020), post-peak period (January 30 ̶ February 13, 2020), and the whole 36-day period (January 9 ̶ February 13, 2020). Each cell in this table presents the estimated median and 95% CrI. Model fitting is performed by fitting a normal distribution via MCMC. Fitting with alternative distributions (e.g., Gumbel distribution) gives similar estimates.

| Factors | Pre-peak period | | | Peak-week period | | | Post-peak period | | | Whole period | | |
| --- | --- | --- | --- | --- | --- | --- | --- | --- | --- | --- | --- | --- |
|  | Mean | sd | n | Mean | sd | n | Mean | sd | n | Mean | sd | n |
| Household | 7.2  (5.9, 8.5) | 5.4  (4.6, 6.5) | 69 | 5.2  (4.5, 6.0) | 5.1  (4.6, 5.7) | 175 | 3.0  (2.0, 3.9) | 4.7  (4.1, 5.5) | 94 | 5.0  (4.4, 5.6) | 5.2  (4.9, 5.7) | 338 |
| Non-household | 8.3  (7.2, 9.3) | 5.1  (4.5, 6.0) | 93 | 5.0  (4.2, 5.7) | 4.9  (4.4, 5.5) | 164 | 2.1  (1.1, 3.1) | 4.5  (3.9, 5.3) | 82 | 5.2  (4.6, 5.8) | 5.3  (4.9, 5.7) | 339 |
| Shorter isolation | 5.3  (4.1, 6.4) | 4.4  (3.7, 5.4) | 60 | 4.1  (3.3, 4.9) | 4.7  (4.2, 5.3) | 140 | 1.6  (0.7, 2.5) | 3.9  (3.4, 4.7) | 77 | 3.3  (2.8, 3.8) | 4.5  (4.1, 4.9) | 292 |
| Longer isolation | 9.4  (8.3, 10.4) | 5.0  (4.3, 5.8) | 91 | 6.0  (5.2, 6.8) | 5.2  (4.7, 5.8) | 168 | 3.4  (2.4, 4.4) | 4.6  (4.0, 5.4) | 80 | 6.8  (6.2, 7.3) | 5.3  (4.9, 5.7) | 324 |
| Younger age | 7.4  (6.3, 8.5) | 4.9  (4.2, 5.8) | 77 | 5.0  (4.3, 5.8) | 4.8  (4.3,5.4) | 159 | 3.2  (2.0, 4.3) | 5.1  (4.4, 6.0) | 80 | 5.1  (4.6, 5.7) | 5.1  (4.7, 5.5) | 316 |
| Older age | 8.8  (7.4, 10.0) | 5.6  (4.8, 6.6) | 74 | 5.2  (4.5, 6.0) | 5.2  (4.7, 5.8) | 175 | 2.1  (1.3, 3.0) | 4.2  (3.7, 4.9) | 94 | 5.1  (4.5, 5.7) | 5.5  (5.1, 5.9) | 343 |
| Male | 7.9  (6.9, 9.0) | 5.3  (4.6, 6.1) | 101 | 5.1  (4.4, 5.8) | 5.3  (4.8, 5.8) | 203 | 2.4  (1.5, 3.2) | 4.5  (3.9, 5.1) | 114 | 5.0  (4.5, 5.6) | 5.4  (5.1, 5.8) | 418 |
| Female | 7.8  (6.5, 9.2) | 5.2  (4.3, 6.3) | 59 | 5.1  (4.3, 5.9) | 4.6  (4.1, 5.2) | 133 | 3.0  (1.7, 4.2) | 4.9  (4.1, 5.9) | 62 | 5.2  (4.6, 5.9) | 5.0  (4.6, 5.5) | 254 |
| All pairs | 7.8  (7.0, 8.6) | 5.2  (4.7, 5.9) | 162 | 5.1  (4.6, 5.7) | 5.0  (4.6, 5.4) | 339 | 2.6  (1.9, 3.2) | 4.6  (4.2, 5.1) | 176 | 5.1  (4.7, 5.5) | 5.3  (5.0, 5.6) | 677 |

Note: SI=Serial interval, sd= Standard deviation, n=size of the transmission pairs

**Table S3**: Estimated mean serial intervals for transmission pairs with infectors developed symptom during the early period (first 14-day running time window during January 9 – 22, 2020) and the end period (last 14-day running time window during January 31 – February 13, 2020). Transmission pairs are stratified by different factors (i.e., isolation delay, age, or gender) and settings (i.e., household, non-household, or all transmission pairs). Each cell in this table presents the estimated median and 95% CrI. Model fitting is performed by fitting a normal distribution to the serial interval data truncated by a 14-day time window. Fitting with alternative distributions (e.g., Gumbel distribution) gives similar estimates.

| **Factors** | | **Household** | | **Non-household** | | **All pairs** | |
| --- | --- | --- | --- | --- | --- | --- | --- |
|  |  | Early period | End period | Early period | End period | Early period | End period |
| **Isolation**  **delay** | Shorter isolation | 4.9  (3.1, 6.7) | 1.9  (0.5, 3.5) | 6.1  (4.5, 7.8) | 0.7  (0.0, 2.0) | 5.3  (4.1, 6.4) | 1.4  (0.5, 2.3) |
|  | Longer isolation | 8.9  (7.1, 10.6) | 3.1  (1.7, 4.5) | 9.6  (8.2, 11.0) | 2.6  (1.0, 4.1) | 9.4  (8.3, 10.4) | 3.0  (1.9, 4.1) |
| **Age** | Younger age | 6.6  (5.2, 8.1) | 3.1  (1.2, 5.0) | 8.0  (6.3, 9.6) | 2.7  (1.2, 4.3) | 7.4  (6.4, 8.6) | 2.9  (1.7, 4.1) |
|  | Older age | 8.2  (5.8, 10.4) | 2.2  (1.0, 3.3) | 9.2  (7.7, 10.7) | 1.0  (0.1, 2.4) | 8.7  (7.5, 10.0) | 1.6  (0.7, 2.5) |
| **Gender** | Male | 7.1  (5.4, 8.8) | 1.7  (0.5, 2.9) | 8.5  (7.2, 9.8) | 2.1  (0.7, 3.5) | 7.9  (6.9, 9.0) | 1.9  (0.9, 2.8) |
|  | Female | 7.6  (5.4, 9.7) | 4.7  (2.5, 6.9) | 8.1  (6.3, 9.9) | 1.5  (0.2, 3.0) | 7.8  (6.5, 9.2) | 2.8  (1.5, 4.1) |
| **All above factors** | | 7.2  (5.9, 8.5) | 2.6  (1.5, 3.6) | 8.3  (7.2, 9.3) | 1.8  (0.7, 2.8) | 7.8  (7.0, 8.6) | 2.2  (1.5, 2.9) |

**Table S4**: Estimated mean serial intervals (with the 95% CI in brackets) from the simulated scenarios under different combinations of isolation delay and initial effective reproduction number$R_{e}$. We approximated the generation time by serial interval distribution with a mean of 2.6 days in scenario-I, 5.1 days in scenario-II, 7.8 days in scenario-III and 8.4 days in scenario-IV.

| Simulated serial intervals (Scenario-I: generation time approximated by serial intervals with mean 2.6 days) | | | | | | | |
| --- | --- | --- | --- | --- | --- | --- | --- |
| Isolation delay (d) | $\boldsymbol{R}_{\boldsymbol{e}}$**=3.0** | $\boldsymbol{R}_{\boldsymbol{e}}$**=2.5** | $\boldsymbol{R}_{\boldsymbol{e}}$**=2.0** | $\boldsymbol{R}_{\boldsymbol{e}}$**=1.5** | $\boldsymbol{R}_{\boldsymbol{e}}$**=1.0** | $\boldsymbol{R}_{\boldsymbol{e}}$**=0.5** | $\boldsymbol{R}_{\boldsymbol{e}}$**=0.3** |
| 0 | 1.6 (0.7, 2.3) | 1.5 (0.8, 2.2) | 1.6 (0.9, 2.2) | 1.6 (1.0, 2.0) | 1.5 (0.9, 2.1) | 1.6 (1.1, 2.1) | 1.5 (1.0, 2.0) |
| 1 | 2.3 (1.5, 2.9) | 2.3 (1.5, 2.9) | 2.2 (1.5, 2.9) | 2.2 (1.5, 2.8) | 2.2 (1.7, 2.8) | 2.3 (1.7, 2.8) | 2.3 (1.7, 2.8) |
| 2 | 2.6 (1.9, 3.2) | 2.5 (1.8, 3.3) | 2.6 (1.9, 3.2) | 2.6 (1.9, 3.2) | 2.6 (2.1, 3.2) | 2.6 (2.1, 3.2) | 2.6 (2.1, 3.1) |
| 3 | 2.6 (1.9, 3.3) | 2.6 (1.8, 3.3) | 2.6 (2.0, 3.3) | 2.6 (2.0, 3.2) | 2.6 (2.1, 3.2) | 2.6 (2.1, 3.1) | 2.6 (2.2, 3.1) |
| 4 | 2.6 (1.8, 3.3) | 2.6 (1.8, 3.3) | 2.6 (1.8, 3.3) | 2.6 (2.0, 3.2) | 2.6 (2.0, 3.2) | 2.6 (2.1, 3.1) | 2.6 (2.1, 3.0) |
| 5 | 2.6 (1.9, 3.2) | 2.6 (1.9, 3.2) | 2.6 (2.0, 3.3) | 2.6 (1.9, 3.2) | 2.6 (2.0, 3.2) | 2.6 (2.1, 3.2) | 2.6 (2.1, 3.1) |
| 6 | 2.6 (1.9, 3.3) | 2.6 (1.9, 3.3) | 2.6 (1.9, 3.1) | 2.6 (2.0, 3.2) | 2.6 (2.0, 3.2) | 2.6 (2.1, 3.2) | 2.6 (2.1, 3.1) |
| 7 | 2.6 (2.0, 3.2) | 2.6 (1.8, 3.3) | 2.6 (1.8, 3.2) | 2.6 (2.0, 3.2) | 2.6 (2.1, 3.3) | 2.6 (2.1, 3.1) | 2.6 (2.1, 3.1) |
| 8 | 2.6 (1.9, 3.3) | 2.6 (1.8, 3.3) | 2.6 (1.9, 3.3) | 2.6 (2.0, 3.2) | 2.6 (2.0, 3.2) | 2.6 (2.1, 3.1) | 2.6 (2.2, 3.2) |
| 9 | 2.6 (1.9, 3.4) | 2.6 (1.9, 3.3) | 2.6 (2.0, 3.3) | 2.6 (1.9, 3.2) | 2.6 (2.0, 3.1) | 2.6 (2.1, 3.2) | 2.6 (2.0, 3.1) |
| 10 | 2.6 (1.8, 3.2) | 2.6 (1.9, 3.3) | 2.6 (1.9, 3.2) | 2.6 (2.0, 3.3) | 2.6 (2.0, 3.1) | 2.6 (1.9, 3.1) | 2.6 (2.1, 3.1) |
| Simulated serial intervals (Scenario-II: generation time approximated by serial intervals with mean 5.1 days) | | | | | | | |
| Isolation delay (d) | $\boldsymbol{R}_{\boldsymbol{e}}$**=3.0** | $\boldsymbol{R}_{\boldsymbol{e}}$**=2.5** | $\boldsymbol{R}_{\boldsymbol{e}}$**=2.0** | $\boldsymbol{R}_{\boldsymbol{e}}$**=1.5** | $\boldsymbol{R}_{\boldsymbol{e}}$**=1.0** | $\boldsymbol{R}_{\boldsymbol{e}}$**=0.5** | $\boldsymbol{R}_{\boldsymbol{e}}$**=0.3** |
| 0 | 1.5 (0.8, 2.2) | 1.5 (0.8, 2.2) | 1.5 (0.7, 2.1) | 1.5 (0.9, 2.1) | 1.5 (1.0, 2.0) | 1.5 (0.9, 1.9) | 1.5 (1.0, 2.0) |
| 1 | 2.3 (1.5, 3.0) | 2.3 (1.7, 3.0) | 2.3 (1.7, 3.0) | 2.3 (1.6, 2.9) | 2.3 (1.8, 2.8) | 2.4 (1.9, 2.9) | 2.3 (1.9, 2.7) |
| 2 | 3.0 (2.4, 3.7) | 3.0 (2.4, 3.5) | 3.0 (2.3, 3.8) | 3.0 (2.4, 3.6) | 3.0 (2.5, 3.6) | 3.1 (2.5, 3.6) | 3.0 (2.5, 3.6) |
| 3 | 3.5 (2.8, 4.3) | 3.6 (2.8, 4.1) | 3.5 (2.8, 4.1) | 3.5 (2.9, 4.2) | 3.6 (3.0, 4.1) | 3.6 (3.0, 4.3) | 3.5 (3.1, 4.0) |
| 4 | 3.9 (3.2, 4.6) | 4.0 (3.3, 4.6) | 4.0 (3.2, 4.5) | 4.0 (3.4, 4.6) | 4.0 (3.4, 4.5) | 4.0 (3.5, 4.5) | 4.0 (3.5, 4.5) |
| 5 | 4.3 (3.5, 5.0) | 4.3 (3.5, 5.0) | 4.3 (3.6, 4.8) | 4.3 (3.7, 4.9) | 4.4 (3.9, 4.9) | 4.3 (3.7, 4.9) | 4.3 (3.8, 4.9) |
| 6 | 4.6 (3.8, 5.3) | 4.5 (3.9, 5.2) | 4.6 (3.8, 5.2) | 4.5 (3.8, 5.2) | 4.5 (3.9, 5.1) | 4.5 (4.0, 5.0) | 4.6 (4.0, 5.1) |
| 7 | 4.7 (3.9, 5.5) | 4.7 (4.0, 5.3) | 4.7 (4.1, 5.3) | 4.7 (4.2, 5.3) | 4.7 (4.1, 5.2) | 4.7 (4.2, 5.2) | 4.7 (4.1, 5.2) |
| 8 | 4.9 (4.1, 5.5) | 4.9 (4.2, 5.5) | 4.9 (4.1, 5.6) | 4.8 (4.1, 5.5) | 4.8 (4.2, 5.3) | 4.9 (4.3, 5.4) | 4.9 (4.3, 5.4) |
| 9 | 4.9 (4.1, 5.6) | 4.9 (4.2, 5.6) | 4.9 (4.3, 5.6) | 5.0 (4.2, 5.6) | 4.9 (4.3, 5.6) | 5.0 (4.4, 5.6) | 4.9 (4.4, 5.5) |
| 10 | 5.1 (4.2, 5.8) | 5.0 (4.2, 5.8) | 5.0 (4.3, 5.6) | 5.0 (4.3, 5.6) | 5.0 (4.4, 5.7) | 5.0 (4.4, 5.6) | 5.0 (4.5, 5.5) |
| Simulated serial intervals (Scenario-III: generation time approximated by serial intervals with mean 7.8 days) | | | | | | | |
| Isolation delay (d) | $\boldsymbol{R}_{\boldsymbol{e}}$**=3.0** | $\boldsymbol{R}_{\boldsymbol{e}}$**=2.5** | $\boldsymbol{R}_{\boldsymbol{e}}$**=2.0** | $\boldsymbol{R}_{\boldsymbol{e}}$**=1.5** | $\boldsymbol{R}_{\boldsymbol{e}}$**=1.0** | $\boldsymbol{R}_{\boldsymbol{e}}$**=0.5** | $\boldsymbol{R}_{\boldsymbol{e}}$**=0.3** |
| 0 | 1.2 (0.3, 2.0) | 1.3 (0.5, 2.1) | 1.3 (0.5, 2.0) | 1.3 (0.7, 2.1) | 1.3 (0.7, 1.9) | 1.3 (0.8, 1.9) | 1.3 (0.6, 2.0) |
| 1 | 2.4 (1.6, 3.1) | 2.4 (1.7, 3.0) | 2.4 (1.8, 3.1) | 2.4 (1.9, 3.0) | 2.5 (1.9, 3.1) | 2.4 (1.9, 2.9) | 2.4 (1.9, 3.1) |
| 2 | 3.6 (2.8, 4.2) | 3.5 (2.8, 4.3) | 3.5 (2.8, 4.2) | 3.5 (2.9, 4.1) | 3.5 (2.8, 4.2) | 3.6 (2.9, 4.1) | 3.5 (2.9, 4.1) |
| 3 | 4.6 (3.8, 5.4) | 4.6 (3.9, 5.3) | 4.6 (3.9, 5.2) | 4.6 (4.0, 5.2) | 4.6 (4.1, 5.2) | 4.6 (4.1, 5.2) | 4.6 (4.1, 5.1) |
| 4 | 5.6 (4.9, 6.3) | 5.6 (4.9, 6.4) | 5.6 (5.0, 6.3) | 5.6 (4.9, 6.2) | 5.6 (5.0, 6.1) | 5.7 (5.1, 6.1) | 5.6 (5.1, 6.1) |
| 5 | 6.5 (5.8, 7.3) | 6.6 (5.8, 7.2) | 6.6 (5.9, 7.2) | 6.6 (5.9, 7.2) | 6.5 (6.0, 7.0) | 6.6 (5.9, 7.1) | 6.6 (6.1, 7.1) |
| 6 | 7.3 (6.6, 8.1) | 7.3 (6.6, 7.9) | 7.3 (6.6, 7.9) | 7.3 (6.8, 7.9) | 7.3 (6.8, 7.9) | 7.3 (6.8, 7.9) | 7.3 (6.8, 7.8) |
| 7 | 7.7 (6.8, 8.4) | 7.8 (7.0, 8.5) | 7.7 (7.1, 8.4) | 7.7 (7.1, 8.2) | 7.7 (7.1, 8.4) | 7.7 (7.1, 8.2) | 7.7 (7.1, 8.2) |
| 8 | 7.8 (7.0, 8.5) | 7.8 (7.0, 8.6) | 7.8 (7.1, 8.5) | 7.8 (7.2, 8.4) | 7.8 (7.2, 8.3) | 7.8 (7.3, 8.3) | 7.8 (7.3, 8.3) |
| 9 | 7.8 (6.9, 8.5) | 7.8 (7.1, 8.5) | 7.8 (7.1, 8.4) | 7.8 (7.2, 8.4) | 7.8 (7.2, 8.3) | 7.8 (7.2, 8.4) | 7.8 (7.3, 8.3) |
| 10 | 7.8 (7.1, 8.5) | 7.8 (7.1, 8.4) | 7.8 (7.0, 8.5) | 7.8 (7.2, 8.3) | 7.8 (7.1, 8.4) | 7.8 (7.3, 8.3) | 7.8 (7.3, 8.4) |
| Simulated serial intervals (Scenario-IV: generation time approximated by serial intervals with mean 8.4 days) | | | | | | | |
| Isolation delay (d) | $\boldsymbol{R}_{\boldsymbol{e}}$**=3.0** | $\boldsymbol{R}_{\boldsymbol{e}}$**=2.5** | $\boldsymbol{R}_{\boldsymbol{e}}$**=2.0** | $\boldsymbol{R}_{\boldsymbol{e}}$**=1.5** | $\boldsymbol{R}_{\boldsymbol{e}}$**=1.0** | $\boldsymbol{R}_{\boldsymbol{e}}$**=0.5** | $\boldsymbol{R}_{\boldsymbol{e}}$**=0.3** |
| 0 | 1.2 (0.4, 2.1) | 1.2 (0.4, 2.0) | 1.3 (0.4, 2.0) | 1.2 (0.5, 1.7) | 1.3 (0.6, 1.9) | 1.2 (0.6, 1.9) | 1.2 (0.5, 2.0) |
| 1 | 2.4 (1.5, 3.1) | 2.3 (1.7, 3.0) | 2.3 (1.6, 3.0) | 2.4 (1.6, 3.0) | 2.4 (1.9, 3.0) | 2.3 (1.7, 2.9) | 2.4 (1.7, 3.1) |
| 2 | 3.4 (2.7, 4.3) | 3.5 (2.7, 4.2) | 3.5 (2.8, 4.2) | 3.5 (2.8, 4.2) | 3.5 (2.9, 4.0) | 3.5 (2.9, 3.9) | 3.4 (2.9, 4.0) |
| 3 | 4.6 (3.8, 5.3) | 4.5 (3.8, 5.2) | 4.6 (4.0, 5.2) | 4.6 (4.0, 5.1) | 4.6 (4.1, 5.1) | 4.5 (4.1, 5.0) | 4.5 (4.0, 5.0) |
| 4 | 5.6 (4.8, 6.3) | 5.7 (5.0, 6.3) | 5.7 (5.0, 6.4) | 5.6 (5.0, 6.2) | 5.6 (5.0, 6.1) | 5.6 (5.0, 6.2) | 5.6 (5.1, 6.1) |
| 5 | 6.6 (5.9, 7.5) | 6.6 (5.8, 7.4) | 6.6 (5.9, 7.2) | 6.6 (6.1, 7.3) | 6.6 (6.1, 7.2) | 6.6 (6.0, 7.1) | 6.6 (6.1, 7.1) |
| 6 | 7.5 (6.8, 8.2) | 7.5 (6.8, 8.1) | 7.5 (6.8, 8.1) | 7.5 (7.0, 8.1) | 7.5 (6.9, 8.1) | 7.5 (7.0, 8.0) | 7.5 (7.1, 8.1) |
| 7 | 8.2 (7.5, 8.9) | 8.1 (7.4, 8.7) | 8.2 (7.4, 8.9) | 8.1 (7.5, 8.7) | 8.1 (7.5, 8.7) | 8.2 (7.6, 8.6) | 8.1 (7.6, 8.7) |
| 8 | 8.4 (7.5, 9.2) | 8.4 (7.7, 9.0) | 8.4 (7.7, 9.1) | 8.4 (7.9, 8.9) | 8.4 (7.8, 9.0) | 8.4 (7.8, 9.0) | 8.4 (7.8, 8.9) |
| 9 | 8.4 (7.6, 9.1) | 8.4 (7.8, 9.1) | 8.4 (7.6, 9.0) | 8.4 (7.8, 9.0) | 8.4 (7.9, 9.0) | 8.4 (7.9, 8.9) | 8.4 (7.8, 9.0) |
| 10 | 8.4 (7.6, 9.1) | 8.4 (7.6, 9.1) | 8.4 (7.8, 9.0) | 8.4 (7.7, 8.9) | 8.4 (7.8, 9.1) | 8.4 (7.8, 8.9) | 8.4 (7.8, 8.9) |

**Table S5**: Proportions of variance in empirical serial intervals explained by the potential factors from the multi-variable regression models.

| Models  (Factors) | Household transmissions | | | Non-household transmissions | | | All transmissions | | |
| --- | --- | --- | --- | --- | --- | --- | --- | --- | --- |
|  | $R^{2}$ | $\%\Delta R^{2}$ | $df$ | $R^{2}$ | $\%\Delta R^{2}$ | $df$ | $R^{2}$ | $\%\Delta R^{2}$ | $df$ |
| Isolation delay^†^ | 0.3859 |  | 23^$^ | 0.4685 |  | 23^$^ | 0.5151 | - | 34^$^ |
| + Isolation of suspected^*^ | 0.4255 | 3.96 | 22 | 0.7663 | 29.78 | 22^$^ | 0.6716 | 15.64 | 33 |
| + Isolation of confirmed^*^ | 0.4269 | 4.10 | 22 | 0.7643 | 29.59 | 22^$^ | 0.6711 | 15.60 | 33 |
| + Inter-city travel ban^*^ | 0.3987 | 1.28 | 22 | 0.7317 | 26.32 | 22^$^ | 0.6473 | 13.22 | 33^$^ |
| + Intra-city travel ban^*^ | 0.4045 | 1.86 | 22 | 0.7278 | 25.93 | 22^$^ | 0.6557 | 14.06 | 33^$^ |
| + Closure of public services^*^ | 0.4267 | 4.08 | 22 | 0.7734 | 30.50 | 22^$^ | 0.6823 | 16.72 | 33 |
| + Closure of entertainment venues^*^ | 0.4257 | 3.98 | 22 | 0.7685 | 30.00 | 22^$^ | 0.6765 | 16.14 | 33 |
| + Social mobilization^*^ | 0.4135 | 2.76 | 22 | 0.7547 | 28.62 | 22^$^ | 0.6724 | 15.73 | 33 |
| + Population immunity^*^ | 0.4545 | 6.86 | 22 | 0.7644 | 29.59 | 22^$^ | 0.7178 | 20.26 | 33 |

† Basic Model: Predicting empirical serial intervals by accounting for isolation delay only.

* Models improved by combining the basic model with each factor.

^$^ Statistically significant (both the coefficients for isolation delay and additional factors)

${\% \Delta R}^{2}$ measures the change in the explained variance from the model in comparison to the basic model.

i.e. $\% {\Delta R}^{2}=(R_{models}^{2}-R_{basic model}^{2})\times100$

1. **References**

1. He X, Lau EHY, Wu P, Deng X, Wang J, Hao X, et al. Temporal dynamics in viral shedding and transmissibility of COVID-19. Nat Med. 2020. Epub 2020/04/17. doi: 10.1038/s41591-020-0869-5. PubMed PMID: 32296168.

2. Bai Y, Yao L, Wei T, Tian F, Jin DY, Chen L, et al. Presumed Asymptomatic Carrier Transmission of COVID-19. JAMA. 2020. Epub 2020/02/23. doi: 10.1001/jama.2020.2565. PubMed PMID: 32083643; PubMed Central PMCID: PMCPMC7042844.

3. Pan X, Chen D, Xia Y, Wu X, Li T, Ou X, et al. Asymptomatic cases in a family cluster with SARS-CoV-2 infection. Lancet Infect Dis. 2020;20(4):410-1. Epub 2020/02/23. doi: 10.1016/S1473-3099(20)30114-6. PubMed PMID: 32087116; PubMed Central PMCID: PMCPMC7158985.

4. Arons MM, Hatfield KM, Reddy SC, Kimball A, James A, Jacobs JR, et al. Presymptomatic SARS-CoV-2 Infections and Transmission in a Skilled Nursing Facility. N Engl J Med. 2020. Epub 2020/04/25. doi: 10.1056/NEJMoa2008457. PubMed PMID: 32329971.

5. Du Z, Xu X, Wu Y, Wang L, Cowling BJ, Meyers LA. Serial Interval of COVID-19 among Publicly Reported Confirmed Cases. Emerg Infect Dis. 2020;26(6). Epub 2020/03/20. doi: 10.3201/eid2606.200357. PubMed PMID: 32191173.

6. Ganyani T, Kremer C, Chen D, Torneri A, Faes C, Wallinga J, et al. Estimating the generation interval for COVID-19 based on symptom onset data. medRxiv. 2020:2020.03.05.20031815. doi: 10.1101/2020.03.05.20031815.

7. Wei WE, Li Z, Chiew CJ, Yong SE, Toh MP, Lee VJ. Presymptomatic Transmission of SARS-CoV-2 - Singapore, January 23-March 16, 2020. MMWR Morb Mortal Wkly Rep. 2020;69(14):411-5. Epub 2020/04/10. doi: 10.15585/mmwr.mm6914e1. PubMed PMID: 32271722; PubMed Central PMCID: PMCPMC7147908 Journal Editors form for disclosure of potential conflicts of interest. No potential conflicts of interest were disclosed.

8. Tindale L, Coombe M, Stockdale JE, Garlock E, Lau WYV, Saraswat M, et al. Transmission interval estimates suggest pre-symptomatic spread of COVID-19. medRxiv. 2020:2020.03.03.20029983. doi: 10.1101/2020.03.03.20029983.

9. Wolfel R, Corman VM, Guggemos W, Seilmaier M, Zange S, Muller MA, et al. Virological assessment of hospitalized patients with COVID-2019. Nature. 2020. Epub 2020/04/03. doi: 10.1038/s41586-020-2196-x. PubMed PMID: 32235945.

10. Huang AT, Garcia-Carreras B, Hitchings MDT, Yang B, Katzelnick L, Rattigan SM, et al. A systematic review of antibody mediated immunity to coronaviruses: antibody kinetics, correlates of protection, and association of antibody responses with severity of disease. medRxiv. 2020:2020.04.14.20065771. doi: 10.1101/2020.04.14.20065771.

11. Rockx B, Kuiken T, Herfst S, Bestebroer T, Lamers MM, Oude Munnink BB, et al. Comparative pathogenesis of COVID-19, MERS, and SARS in a nonhuman primate model. Science. 2020. Epub 2020/04/19. doi: 10.1126/science.abb7314. PubMed PMID: 32303590; PubMed Central PMCID: PMCPMC7164679.

12. Li Q, Guan X, Wu P, Wang X, Zhou L, Tong Y, et al. Early Transmission Dynamics in Wuhan, China, of Novel Coronavirus-Infected Pneumonia. N Engl J Med. 2020;382(13):1199-207. Epub 2020/01/30. doi: 10.1056/NEJMoa2001316. PubMed PMID: 31995857.

13. Bi Q, Wu Y, Mei S, Ye C, Zou X, Zhang Z, et al. Epidemiology and transmission of COVID-19 in 391 cases and 1286 of their close contacts in Shenzhen, China: a retrospective cohort study. Lancet Infect Dis. 2020. Epub 2020/05/01. doi: 10.1016/S1473-3099(20)30287-5. PubMed PMID: 32353347; PubMed Central PMCID: PMCPMC7185944.

14. Zhang J, Litvinova M, Wang W, Wang Y, Deng X, Chen X, et al. Evolving epidemiology and transmission dynamics of coronavirus disease 2019 outside Hubei province, China: a descriptive and modelling study. Lancet Infect Dis. 2020. Epub 2020/04/06. doi: 10.1016/S1473-3099(20)30230-9. PubMed PMID: 32247326.

15. Leung K, Wu JT, Liu D, Leung GM. First-wave COVID-19 transmissibility and severity in China outside Hubei after control measures, and second-wave scenario planning: a modelling impact assessment. Lancet. 2020. Epub 2020/04/12. doi: 10.1016/S0140-6736(20)30746-7. PubMed PMID: 32277878.

16. Tian H, Liu Y, Li Y, Wu CH, Chen B, Kraemer MUG, et al. An investigation of transmission control measures during the first 50 days of the COVID-19 epidemic in China. Science. 2020. Epub 2020/04/03. doi: 10.1126/science.abb6105. PubMed PMID: 32234804; PubMed Central PMCID: PMCPMC7164389.
